# Withaninsams A and B: Phenylpropanoid Esters from the Roots of Indian Ginseng (*Withania somnifera*)

**DOI:** 10.3390/plants8120527

**Published:** 2019-11-20

**Authors:** Su Cheol Baek, Seoyoung Lee, Sil Kim, Mun Seok Jo, Jae Sik Yu, Yoon-Joo Ko, Young-Chang Cho, Ki Hyun Kim

**Affiliations:** 1 School of Pharmacy, Sungkyunkwan University, Suwon 16419, Korea; schii513@daum.net (S.C.B.); malin_1272@naver.com (S.K.); anstjr920827@gmail.com (M.S.J.); jsyu@bu.edu (J.S.Y.); 2 College of Pharmacy, Chonnam National University, Gwangju 61186, Korea; 96_29@naver.com (S.L.); yccho@jnu.ac.kr (Y.-C.C.); 3 Laboratory of Nuclear Magnetic Resonance, National Center for Inter-University Research Facilities (NCIRF), Seoul National University, Gwanak-gu, Seoul 08826, Korea; yjko@snu.ac.kr

**Keywords:** *Withania somnifera*, phenylpropanoid esters, Withaninsams A and B, nitric oxide, inducible nitric oxide synthase

## Abstract

*Withania somnifera* (L.) Dunal (Solanaceae), known as Indian ginseng or ashwagandha, has been used in Indian Ayurveda for the treatment of a variety of disorders, such as diabetes and reproductive and nervous system disorders. It is particularly used as a general health tonic, analgesic, and sedative. As part of continuing projects to discover unique bioactive natural products from medicinal plants, phytochemical investigation of the roots of *W. somnifera* combined with a liquid chromatography–mass spectrometry (LC/MS)-based analysis has led to the isolation of two novel phenylpropanoid esters, Withaninsams A (**1**) and B (**2**), as an inseparable mixture, along with three known phenolic compounds (**3**, **4**, and **6**) and a pyrazole alkaloid (**5**). The structures of the new compounds were elucidated using a combination of spectroscopic methods, including one-dimensional (1D) and two-dimensional (2D) nuclear magnetic resonance (NMR) and high-resolution electrospray ionization mass spectroscopy (HR-ESIMS). Withaninsams A (**1**) and B (**2**) are phenylpropanoid esters that contain a side chain, 4-methyl-1,4-pentanediol unit. To the best of our knowledge, the present study is the first to report on phenylpropanoid esters with 4-methyl-1,4-pentanediol unit. The anti-inflammatory activity of the isolated compounds (**1**–**6**) was evaluated by determining their inhibitory effects on nitric oxide (NO) production in lipopolysaccharide (LPS)-stimulated RAW 264.7 macrophages, where compound 3 inhibited LPS-induced NO production (IC_50_ = 33.3 μM) and TNF-α production, a pro-inflammatory cytokine (IC_50_ = 40.9 μM). The anti-inflammatory mechanism through the inhibition of transcriptional iNOS protein expression was confirmed by western blotting experiments for the active compound **3**, which showed decreased iNOS protein expression.

## 1. Introduction

*Withania somnifera* (L.) Dunal (Solanaceae), commonly known as Indian ginseng or ashwagandha, is a perennial shrub distributed in India, Morocco, Egypt, Israel, Jordan, South Africa, and the Mediterranean region. Currently, it is cultivated on a small scale in South Korea as well [1,2,3]. This plant has been used in Indian Ayurveda for over 3000 years for the treatment of a variety of disorders, such as diabetes and nervous and reproductive disorders. It is particularly used as a general health tonic, analgesic, and sedative [4]. The name, Indian ginseng is botanically not related to Korean ginseng (*Panax ginseng*). The similarity in the name arises from its similar bioactivity [5]. Roots of *W. somnifera* have recently gained popularity as a functional food that promotes longevity through delaying aging, increasing immunity against extrinsic factors, and strengthening the body [1,6]. In practice, extracts of the roots are consumed in several forms, such as powder, liquid, tablets, and capsules. Moreover, roots are consumed as a dietary supplement.

Previous pharmacological studies on *W. somnifera* have revealed that extracts of *W. somnifera* exhibit a protective role against bromobenzene-induced oxidative damage in the rat liver [7], as well as increase the exercise performance [8]. Similarly, a recent study on *W. somnifera* extracts reported its therapeutic role in stroke repair through its anti-apoptotic and anti-oxidant properties [9]. Chemically, it is a rich source of withanolides, which possess diverse pharmacological properties, including anti-inflammatory, anti-microbial, anti-tumor, hepatoprotective, and immunosuppressive effects [10,11,12,13,14,15,16]. In addition, alkaloids, steroidal saponins, lignanamides [17], and phenolics [18] have also been reported, and some of them were found to have anti-tumor activities [19].

**Figure 1:**
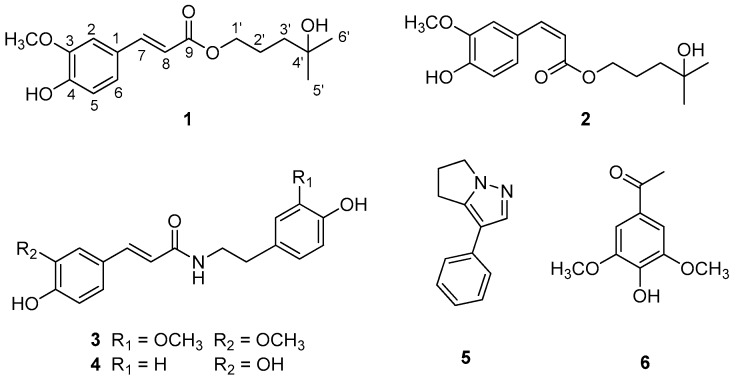
Chemical structures of compounds **1**–**6**.

As part of a continuing program to determine structurally and/or biologically novel natural products from medicinal plants [20,21,22,23], we conducted a chemical investigation of the methanol (MeOH) extract of roots of *W. somnifera*. In our recent study, chemical analysis of the MeOH extract, combined with a liquid chromatography/mass spectroscopy (LC/MS)-based analysis, we identified six new withanolides, namely withasilolides A to F, and seven known withanolides [3], some of which exhibited cytotoxicity against several human cancer cell lines (A549, SK-OV-3, SK-MEL-2, and HCT-15). In the present study, we focused on other constituents of *W. somnifera* rather than withanolides. The phytochemical analysis of the MeOH extract led to the isolation of two new phenylpropanoid esters (**1** and **2**) as an inseparable mixture, along with four known compounds (**3**–**6**) (Figure 1). The structures of the new compounds (**1** and **2**) were elucidated using a combination of one-dimensional (1D) and two-dimensional (2D) nuclear magnetic resonance (NMR) spectroscopy and high-resolution electrospray ionization mass spectroscopy (HR-ESIMS) data. Further, we evaluated the inhibitory effects of the isolates on nitric oxide (NO) production in lipopolysaccharide (LPS)-activated RAW 264.7 macrophages. In the present study, we describe the isolation and structural characterization of isolated compounds, as well as the evaluation of their NO inhibitory effects on LPS-activated RAW264.7 macrophages.

## 2. Results and Discussion

### 2.1. Isolation of Compounds

The dried roots of *W. somnifera* were extracted with 80% MeOH under reflux to yield the methanol extract, which was sequentially applied to solvent-partitioning with hexane, dichloromethane, ethyl acetate, and *n*-butanol to obtain each solvent fraction. Chemical analysis of hexane-soluble and dichloromethane-soluble fractions was performed using repeated column chromatography and high-performance liquid chromatography (HPLC) along with LC/MS-based analysis. These techniques were combined with our house-built UV library to determine other types of minor constituents, rather than withanolides. The analysis led to the isolation of two new phenylpropanoid esters (**1** and **2**) as an inseparable mixture, along with three known phenolic compounds (**3**, **4**, and **6**) and a pyrazole alkaloid (**5**) (Figure 1). The novel compounds resulted in only one peak in HPLC performed using Phenomenex Luna C18 column (MeOH/H_2_O, 7:3 to 1:0).

### 2.2. Structure Elucidation of Compounds

**Figure 2:**
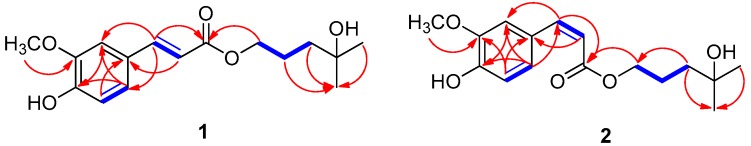
^1^H-^1^H correlation spectroscopy (COSY) (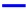) and key heteronuclear multiple bond correlation (HMBC) (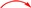) correlations for compounds **1** and **2**.

Compounds **1** and **2** were obtained as a *trans/cis* inseparable mixture in the ratio of approximately 5:4 (as calculated from the ^1^H NMR integral). They were isolated as an amorphous powder, and their HR-ESIMS (Figure S1) showed the molecular ion peak [M + Na]^+^ at *m/z* 317.1375 (calculated for C_16_H_22_O_5_Na, 317.1365) in the positive mode, compatible with the molecular formula of C_16_H_22_O_5_. The IR spectrum exhibited characteristic absorptions of hydroxy (3305 cm^−1^) and ester groups (1755 cm^−1^). The ^1^H NMR spectrum (Table 1, Figure S2) showed complicated aromatic proton signals in the narrow region between *δ*_H_ 6.77 and 7.08. Most of the ^13^C NMR signals, which were assigned by ^1^H-^1^H correlation spectroscopy (COSY) (Figure S3), heteronuclear single quantum coherence (HSQC) (Figure S4), and heteronuclear multiple bond correlation (HMBC) (Figure S5) experiments, were split into pairs of narrowly separated signals (Table 1). Based on the assigned molecular formula (C_16_H_22_O_5_) by HR-ESIMS, we deduced that the two compounds could be a mixture of two closely related compounds, **1** and **2**. The detailed inspection of the ^1^H NMR spectrum (Table 1) revealed the presence of two sets of 1,3,4-trisubstituted aromatic protons, one at δ_H_ 6.89 (1H, d, *J* = 8.0 Hz), 7.01 (1H, d, *J* = 1.5 Hz), and 7.05 (1H, dd, *J* = 8.0, 1.5 Hz) and another at δ_H_ 6.86 (1H, d, *J* = 8.0 Hz), 7.08 (1H, dd, *J* = 8.0, 2.0 Hz), and 7.74 (1H, d, *J* = 2.0 Hz), as well as two methoxy groups at δ_H_ 3.91 (6H, s). Interpretation of the ^1^H NMR and ^1^H-^1^H COSY spectra (Figure S3) led to the following structural units: C-5 to C-6 and C-7 to C-8 (Figure 2). Connectivities of the partial structure of phenylpropanoid were established on the HMBC cross-peaks of H-2/C-4, C-6, H-5/C-1, C-3, H-6/C-2, C-4, H-7/C-2, C-6, C-9, and H-8/C-1, C-9 (Figure 2). The HMBC correlation of the methoxy group (δ_H_ 3.91)/C-3 provided evidence of the presence of the methoxy group at C-3. The presence of 4-methyl-1,4-pentanediol was established on the HMBC cross-peaks of H-1’/C-3’, H-3’/C-1’, C-5’, C-6’, H-5’/C-3’, C-6’, and H-6’/C-3’, C-5’, together with ^1^H-^1^H COSY correlations from H-1’ to H-3’ (Figure 2). The linkage between the phenylpropanoid and 4-methyl-1,4-pentanediol unit was established using the HMBC correlation from H-1’ to C-9 (Figure 2).

The geometry of the di-substituted olefin Δ^7/8^ in compounds **1** and **2** was determined on the basis of the vicinal ^1^H coupling constant (*J*_7,8_ = 16.0 Hz in **1**; *J*_7,8_ = 13.0 Hz in **2**) [24], which indicated that compound **1** had *trans*-conformation, whereas compound 2 had *cis*-conformation. Moreover, this assignment is supported by the typical NMR chemical shifts exhibiting δ_H_6.27 (H-8) and δ_H_7.58 (H-7) in compound **1** (*trans*) and δ_H_5.79 (H-8) and δ_H_6.77 (H-7) in compound **2** (*cis*) [24]. Therefore, structures of compounds **1** and **2** were determined as shown in Figure 1, with their trivial names designated as Withaninsams A (**1**) and B (**2**), respectively. Withaninsams A (**1**) and B (**2**) are phenylpropanoid esters containing a side chain, 4-methyl-1,4-pentanediol unit, which is a rare example of a natural product. Although phenylpropanoid esters with similar side chains, such as *tert*-butyl *trans*-ferulate and *tert*-butyl (2*E*)-3-(3,4-dimethoxyphenyl)-2-propenoate, have been reported [25,26], to the best of our knowledge, the present study is the first to report on phenylpropanoid esters with 4-methyl-1,4-pentanediol unit. Finally, we attempted to separate the mixture of **1** and **2** using the chiral HPLC column, Phenomenex Lux Cellulose-1; however, we failed to resolve the mixture into a pair of pure enantiomers under different conditions.

The known compounds were identified as *N*-*trans*-feruloyl methoxytyramine (**3**) [27], *N*-*trans*-feruloyltyramine (**4**) [28], withasomnine (**5**) [29], and acetosyringone (**6**) [30] by comparing their NMR spectroscopic data with reported values as well as using the LC/MS analysis results. 

### 2.3. Inhibitory Effects of Compounds ***1**–**6*** on LPS-Induced NO Production in RAW 264.7 Cells

**Figure 3:**
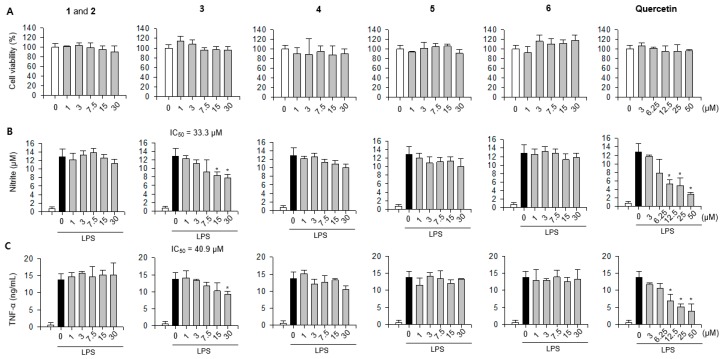
Inhibitory effects of compounds **1**–**6** on the production of pro-inflammatory mediators in macrophages. RAW 264.7 cells were treated with compounds **1**–**6** in the absence or presence of lipopolysaccharide (LPS) and then (**A**) cell viability, (**B**) nitric oxide (NO) production, and (**C**) TNF-α production in RAW 264.7 cells were determined. Quercetin was used as a positive control. (**A**) Cell viability data represent relative cell viability compared with that of the untreated group (100%, white bar). (**B**,**C**) NO and TNF-α production data represent actual production levels calculated by applying absorbance values to each standard curve. (white bar, untreated control group; grey and black bar, LPS-treated control group). *p* < 0.05 relative to the LPS-treated control group.

**Figure 4:**
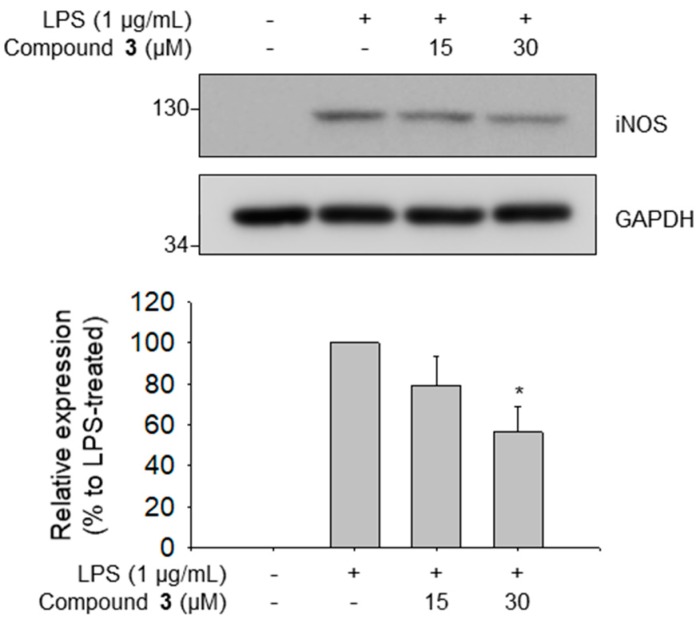
Inhibitory effect of compound **3** on LPS-induced nitric oxide synthase (iNOS) expression in RAW 264.7 cells. Data represent relative iNOS protein expression levels compared with those in the LPS-treated group (100%). *p* < 0.05 relative to the LPS-treated control group.

To determine whether the isolated compounds **1** to **6** had anti-inflammatory properties, we performed NO assay using supernatants from LPS-stimulated RAW 264.7 cells. All compounds tested exhibited no cytotoxicity up to each highest concentration (Figure 3A). Of these, compound **3** exhibited an inhibitory effect on NO production in LPS-stimulated RAW 264.7 cells (IC_50_ = 33.3 μM) (Figure 3B). The effect of compound **3** on the LPS-induced TNF-α production showed similar patterns to those of NO production (IC_50_ = 40.9 μM) (Figure 3C). To confirm whether NO inhibitory effect of 3 was related to reduced expression of nitric oxide synthase (iNOS), enzyme involved in the synthesis of NO, we evaluated the effect of compound **3** on iNOS expression. Similar to the results of NO production, compound **3** reduced iNOS expression in LPS-stimulated RAW 264.7 cells (Figure 4). These results indicated that compound **3** exerted anti-inflammatory effect on macrophages by reducing LPS-induced NO production through transcriptional inhibition of iNOS. 

## 3. Materials and Methods

### 3.1. Plant Material

The roots of *W. somnifera* were purchased from Seonggeosan Farm, Cheonan, Korea, in October 2016, and the plant was identified by one of the authors (K.H.K.). A voucher specimen of the material (IDG-2016) was deposited in the herbarium of the School of Pharmacy, Sungkyunkwan University, Suwon, Republic of Korea.

### 3.2. Extraction and Isolation

Dried roots of *W. somnifera* (1.3 kg) were extracted with 80% aqueous MeOH (each 3.0 L × 3 days) under reflux and filtered. The filtrate was combined and concentrated under vacuum using a rotary evaporator to obtain a MeOH extract (189.6 g). The extract was suspended in distilled water (700 mL) and successively solvent-partitioned using hexane, dichloromethane (CH_2_Cl_2_), ethyl acetate (EtOAc), and *n*-butanol (*n*-BuOH). Four fractions with increasing polarity were obtained: hexane-soluble (3.4 g), CH_2_Cl_2_-soluble (4.5 g), EtOAc-soluble (2.0 g), and *n*-BuOH-soluble (18.6 g). The hexane-soluble fraction (3.2 g) was fractionated using silica gel column chromatography with a gradient solvent system of hexane/EtOAc (20:1 to 1:1). The column was washed with CH_2_Cl_2_/MeOH (1:1) to obtain seven hexane fractions (H1–H7). Fraction H6 (300.1 mg) was separated by preparative reversed-phase HPLC using an Agilent Eclipse C_18_ column with gradient solvent system MeOH/H_2_O (7:3 to 1:0) to obtain seven subfractions (H6a–H6g). Subfraction H6c (134.6 mg) was purified by semi-preparative HPLC (38% MeCN) on Phenomenex Luna C18 column to yield compound **5** (24.3 mg). Subfraction H6g (22.9 mg) was purified by semi-preparative HPLC with gradient solvent system MeOH/H_2_O (7:3 to 1:0) on Phenomenex Luna C18 column to yield a mixture of compounds **1** and **2** (1.1 mg). The CH_2_Cl_2_-soluble fraction (4.5 g) was fractionated using silica gel column chromatography with gradient solvent system of CH_2_Cl_2_/MeOH (50:1 to 1:1) to obtain seven fractions (C1–C7). Fraction C1 (73.6 mg) was separated by preparative reversed-phase HPLC on an Agilent Eclipse C_18_ column with gradient solvent system MeOH/H_2_O (3:7 to 1:0) to obtain five subfractions (C1a–C1e). Subfraction C1c (11.8 mg) was purified using semi-preparative HPLC (33% MeOH) on Phenomenex Luna C18 column to yield compound **6** (7.2 mg). Fraction C3 (401.6 mg) was fractionated using preparative reversed-phase HPLC on Agilent Eclipse C_18_ column with gradient solvent system MeOH/H_2_O (3:7 to 1:0) to obtain six subfractions (C3a–C3f). Subfraction C3d (33.8 mg) was purified using semi-preparative HPLC on Phenomenex Luna C18 column (47% MeOH) to yield compound **3** (2.4 mg). Fraction C4 (166.0 mg) was separated by reversed-phase preparative HPLC using an Agilent Eclipse C_18_ column with gradient solvent system MeOH/H_2_O (4:6 to 9:1) to obtain six subfractions (C4a–C4f). Subfraction C4c (19.2 mg) was purified by semi-preparative HPLC (40% MeOH) on Phenomenex Luna C18 column to yield compound **4** (3.3 mg).

#### Withaninsams A (**1**) and B (**2**)

White, amorphous powder; ESIMS (positive mode) *m/z:*317 [M + Na]^+^; HRESIMS (positive mode) *m/z:*317.1375 [M + Na]^+^, calcd for C_16_H_22_O_5_Na, 317.1365; UV (MeOH) λ_max_ nm (log ε): 220 (2.29), 240 (2.10), 290 (2.54), 325 (3.42); IR (KBr) *ν*_max_ cm^−1^: 3305, 3126, 1755, 1585, 1492, 1042; ^1^H (CD_3_OD, 800 MHz) and ^13^C (CD_3_OD, 200 MHz) NMR spectroscopic data, see Table 1.

### 3.3. Cell Viability Assay

RAW 264.7 cells (6.0 × 10^4^ cells/well) were seeded into a 96-well plate and incubated overnight for adhesion. Following incubation, the cells were treated with compounds for 24 h. Next, Ez-CytoX solution (1/10 volume of the culture medium, Daeil Lab., Seoul, Korea) was added to each well and cells were further incubated for 1 h. The cell viability was assessed by measuring the absorbance at 450 nm.

### 3.4. NO Production Assay

RAW 264.7 cells (6.0 × 10^4^ cells/well) were seeded into a 96-well plate and incubated overnight for adhesion. Following incubation, the cells were treated with compounds and LPS. After 24 h incubation, supernatants were collected and treated with Griess reagent for evaluating NO concentration in the reactants. The absorbance was measured at 540 nm and NO production was calculated by referring to the nitrite standard curve.

### 3.5. TNF-α ELISA

Culture supernatants were applied to ELISA experiments for measuring the production of TNF-α in LPS-stimulated RAW 264.7 cells. ELISA was performed by manufacturer’s instructions (Ebioscience, San Diego, CA, USA). Each step was followed by washing with 1 × PBST 5 times. Briefly, plate was coated with coating antibody solution overnight at 4 °C and then blocked with 1 × assay diluent for 1 h at room temperature (RT). Supernatants were applied to the plate for 2 h at RT and the plate was incubated with biotinylated secondary antibody for 1 h at RT. After reacting the plate with horseradish peroxidase (HRP)-streptavidin for 40 min at RT, the plate was reacted with 4-nitrophenyl phosphate disodium salt in diethanolamine buffer as a substrate for 10 min at dark condition and the reaction was stopped by adding 1 N NaOH. Absorbances of each well at 405 nm were applied to standard curve for calculating the quantity of TNF-α at supernatants.

### 3.6. Western Blotting

RAW 264.7 cells (2.0 × 10^5^ cells/well) were seeded into 6-well plates and incubated overnight for adhesion. Following incubation, the cells were treated with compounds **3** and **4** in the presence of LPS for 24 h. Total cell lysates were obtained and loaded onto sodium dodecyl sulfate-polyacrylamide gel electrophoresis (SDS-PAGE). Proteins were subsequently transferred onto nitrocellulose (NC) membrane. Membranes were incubated with primary antibodies against iNOS and GAPDH, following which the membranes were incubated with appropriate secondary antibodies. Finally, the membranes were blotted and protein band intensities were analyzed using Imager 680 (GE Healthcare; Chicago, IL, USA).

### 3.7. Statistical Analysis

The data were statistically analyzed using Student’s *t*-test. To prove statistical significance, experiments were conducted in replicates as follows: nine for cell viability, NO assay, TNF-α ELISA and three for western blotting. A *p-*value < 0.05 was considered statistically significant. 

## 4. Conclusions

In the present study, phytochemical analysis of the MeOH extracts of roots of *W. somnifera* led to the isolation of two novel phenylpropanoid esters, namely Withaninsams A (**1**) and B (**2**) as an inseparable mixture, along with three known phenolic compounds (**3**, **4**, and **6**) and a pyrazole alkaloid (**5**). Withaninsams A (**1**) and B (**2**) are phenylpropanoid esters that contain a side chain, 4-methyl-1,4-pentanediol unit. To the best of our knowledge, the present study is the first to report on phenylpropanoid esters with 4-methyl-1,4-pentanediol unit. All isolated compounds were evaluated for their anti-inflammatory effects on nitric oxide (NO) production in LPS-stimulated RAW 264.7 macrophages. Compound **3** exhibited NO and TNF-α inhibitory properties without cytotoxicity. The active compound **3** inhibited NO production by reducing iNOS protein expression.

## Figures and Tables

**Table 1:** ^1^H and ^13^C NMR data of compounds **1** and **2** in CD_3_OD (δ in ppm, 800 MHz for ^1^H and 200 MHz for ^13^C) ^a^.

Position	1		2	
	*δ*_H_	*δ*_C_	*δ*_H_	*δ*_C_
1		127.0		127.3
2	7.01 d (1.5)	109.2	7.74 d (2.0)	112.6
3		146.5		145.9
4		147.9		147.0
5	6.89 d (8.0)	114.6	6.86 d (8.0)	113.7
6	7.05 dd (1.5, 8.0)	123.1	7.08 dd (2.0, 8.0)	125.5
7	7.58 d (16.0)	114.6	6.77 d (13.0)	143.7
8	6.27 d (16.0)	115.6	5.79 d (13.0)	116.8
9		167.3		166.7
1′	4.16 t (7.0)	64.4	4.09 t (7.0)	64.4
2′	1.37 m	28.6	1.30 m	28.5
3′	1.67 m	25.9	1.62 m	25.8
4′		n.d.^b^		n.d. ^b^
5′	1.23 s	29.5	1.23 s	29.5
6′	1.23 s	29.5	1.23 s	29.5
–OCH_3_	3.91 s	55.6	3.91 s	55.6

^a^*J* values are in parentheses and reported in Hz; ^13^C NMR assignments are based on ^1^H-^1^H COSY, HSQC, and HMBC experiments; ^b^ not detected.

## Supplementary Materials

The following are available online at https://www.mdpi.com/2223-7747/8/12/527/s1, Figure S1: The HRESIMS data of compounds **1** and **2**, Figure S2: The ^1^H NMR spectrum of compounds **1** and **2**, Figure S3: The ^1^H-^1^H COSY spectrum of compounds **1** and **2**, Figure S4: The HSQC spectrum of compounds **1** and **2**, Figure S5: The HMBC spectrum of compounds **1** and **2**.
