# Supplementary material for: Withaninsams A and B: Phenylpropanoid Esters from the Roots of Indian Ginseng (Withania somnifera)
Source: Plants (Basel). 2019 Nov 20;8(12):527. doi: 10.3390/plants8120527 (PMC6963882; doi:10.3390/plants8120527)
Supplement: Supplementary file 1 [file plants-08-00527-s001.pdf]

plants

MDPI

Supplementary Materials

Supporting Information Contents:

General experimental procedures …………………………………………………………………………………………S3
Figure S1. The HRESIMS data of compounds **1** and **2**……………………………………………………………………S4
Figure S2. The ^{1}H NMR spectrum of compounds **1** and **2** (CD_3OD, 800 MHz)………… …………………………S5
Figure S3. The ^{1}H-^{1}H COSY spectrum of compounds **1** and **2**………………………………………………S6
Figure S4. The HSQC spectrum of compounds **1** and **2**……………………………………………………………………S7
Figure S5. The HMBC spectrum of compounds **1** and **2**……………………………………………………………………S8

General experimental procedures

Optical rotations were measured using a JASCO P-2000 polarimeter (JASCO, Easton, MD, USA). Ultraviolet spectra were acquired on an Agilent 8453 UV−visible spectrophotometer (Agilent Technologies, Santa Clara, CA, USA). Infrared spectra were recorded with a Bruker IFS-66/S FT-IR spectrometer (Bruker, Karlsruhe, Germany). NMR spectra were recorded with a Bruker AVANCE III HD 800 NMR spectrometer with a 5 mm TCI CryoProbe operating at 800 MHz (^{1}H) and 200 MHz (^{13}C), with chemical shifts given in ppm (δ) for ^{1}H and ^{13}C NMR analyses. All HRESIMS data were obtained with a Waters Xevo G2 QTOF mass spectrometer and Synapt G2 HDMS quadrupole time-of-flight (TOF) mass spectrometer (Waters). Preparative high-performance liquid chromatography (HPLC) was performed using a Waters 1525 Binary HPLC pump with a Waters 996 photodiode array detector (Waters Corporation, Milford, MA, USA) and an Agilent Eclipse C_{18} column (250 × 21.2 mm, 5 µm; flow rate: 5 mL/min; Agilent Technologies). Semipreparative HPLC was performed using a Shimadzu Prominence HPLC System with SPD-20A/20AV Series Prominence HPLC UV−vis detectors (Shimadzu, Tokyo, Japan) and a Phenomenex Luna C18 column (250 × 10 mm, 5 µm; flow rate: 2 mL/min; Phenomenex, Torrance, CA, USA). LC/MS analysis was performed on an Agilent 1200 Series HPLC system equipped with a diode array detector and 6130 Series ESI mass spectrometer using an analytical Kinetex C18 100 Å column (100 × 2.1 mm, 5 µm; flow rate: 0.3 mL/min; Phenomenex). Silica gel 60 (230−400 mesh, Merck, Darmstadt, Germany) and RP-C_{18} silica gel (Merck, 230−400 mesh) were used for column chromatography. The packing material for molecular sieve column chromatography was Sephadex LH-20 (Pharmacia, Uppsala, Sweden). Thin-layer chromatography (TLC) was performed with precoated silica gel F254 plates and RP-C_{18} F254s plates (Merck), and spots were detected under UV light or by heating after spraying with anisaldehyde-sulfuric acid.

Single Mass Analysis
Tolerance = 5.0 mDa / DBE: min = -1.5, max = 50.0
Element prediction: Off
Number of isotope peaks used for i-FIT = 3
Monoisotopic Mass, Even Electron Ions
107 formula(e) evaluated with 3 results within limits (up to 50 closest results for each mass)
Elements Used:

Mass	Calc. Mass	mDa	PPM	DBE	Formula	i-FIT	i-FIT Norm	Fit Conf %	C	H	O	Na
317.1375	317.1365	1.0	3.2	5.5	C16 H22 O5 Na	292.8	0.456	63.39	16	22	5	1
	317.1389	-1.4	-4.4	8.5	C18 H21 O5	293.5	1.140	31.99	18	21	5	
	317.1330	4.5	14.2	17.5	C25 H17	295.4	3.074	4.63	25	17		

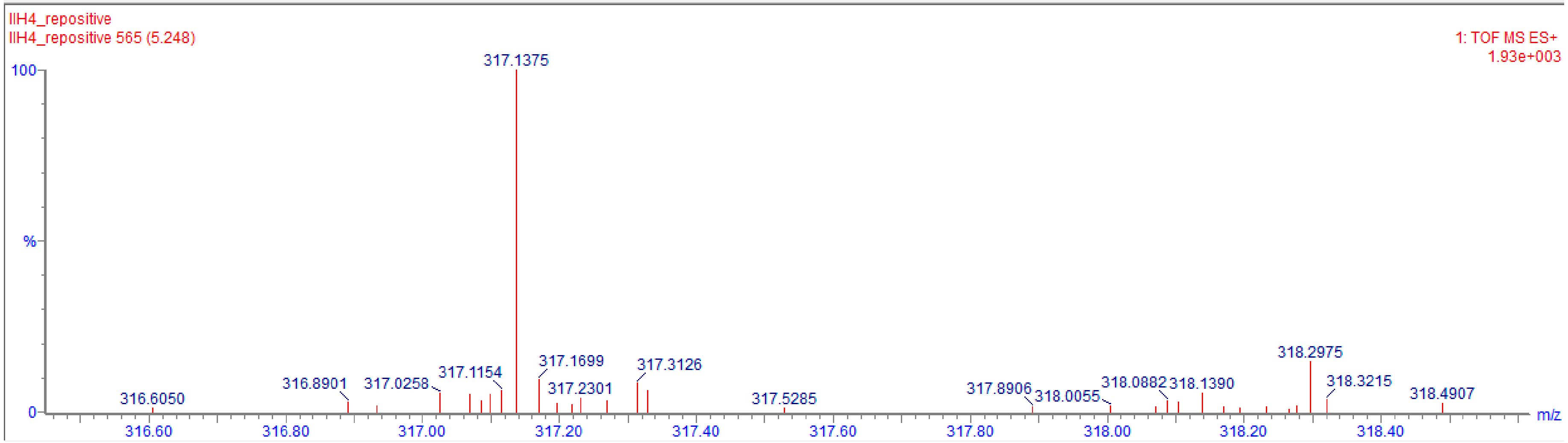

Figure S1. The HRESIMS data of compounds **1** and **2.**

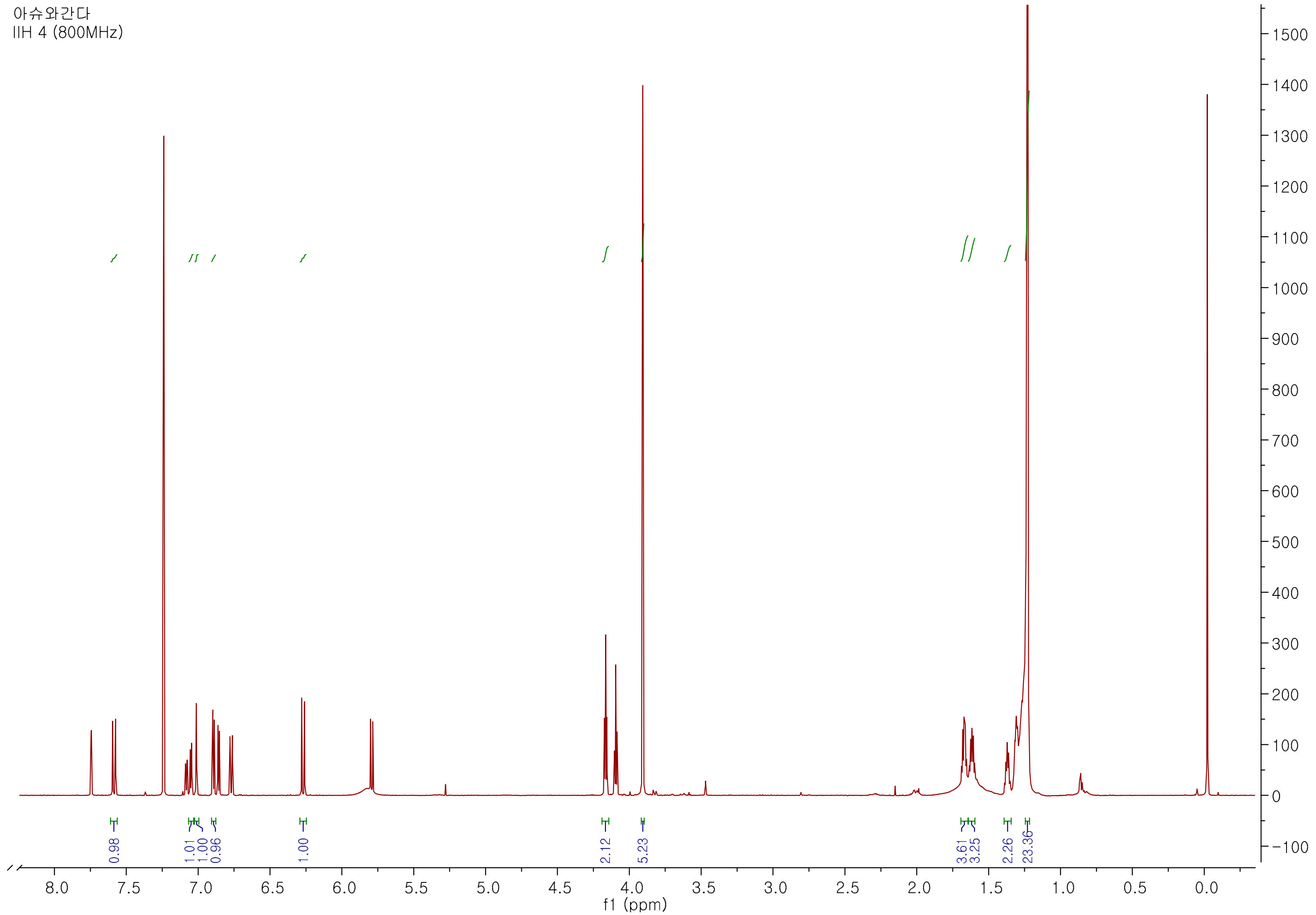

Figure S2. The ^{1}H NMR spectrum of compounds **1** and **2** (CD_3OD, 800 MHz).

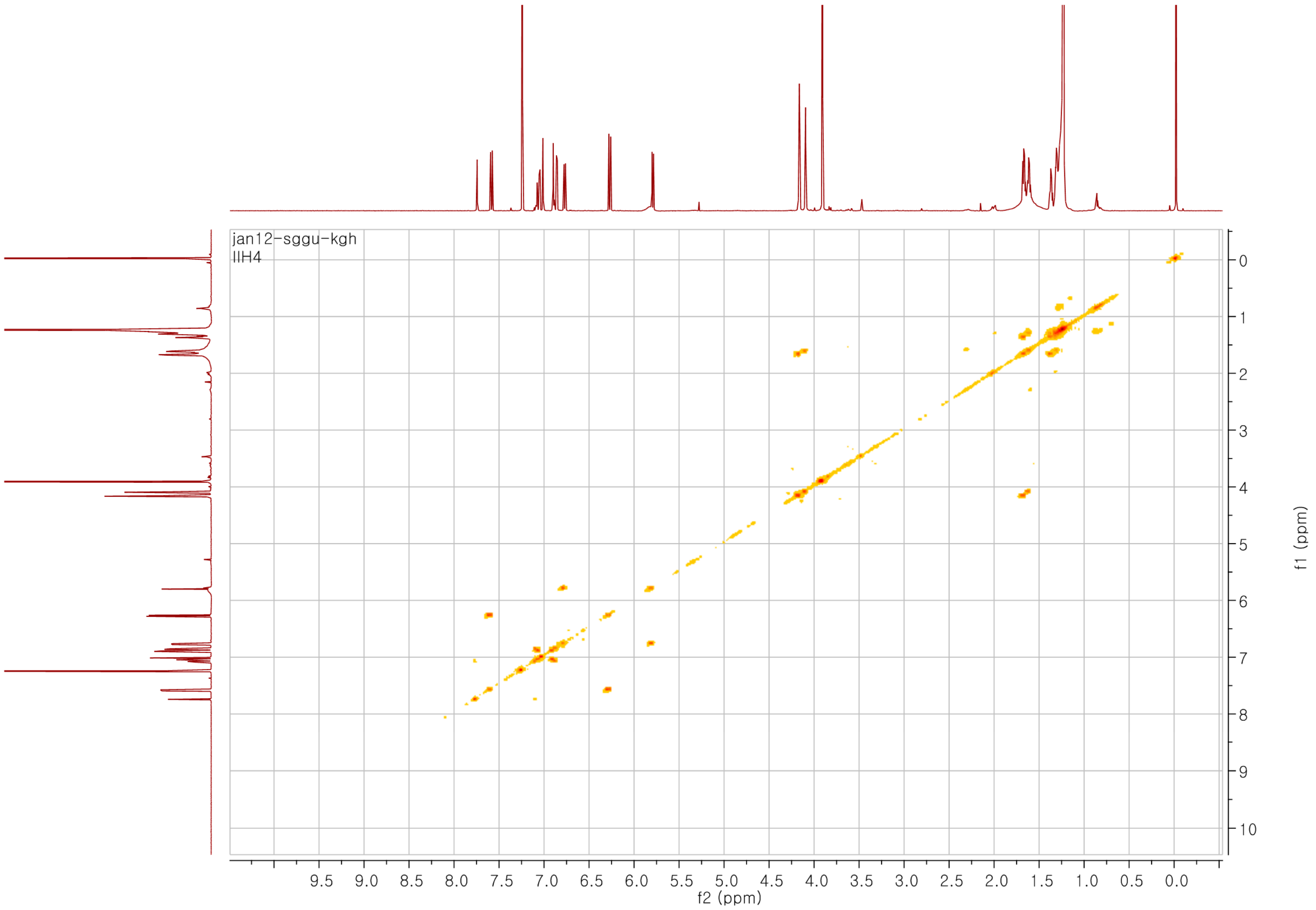

Figure S3. The ^{1}H-^{1}H COSY spectrum of compounds **1** and **2.**

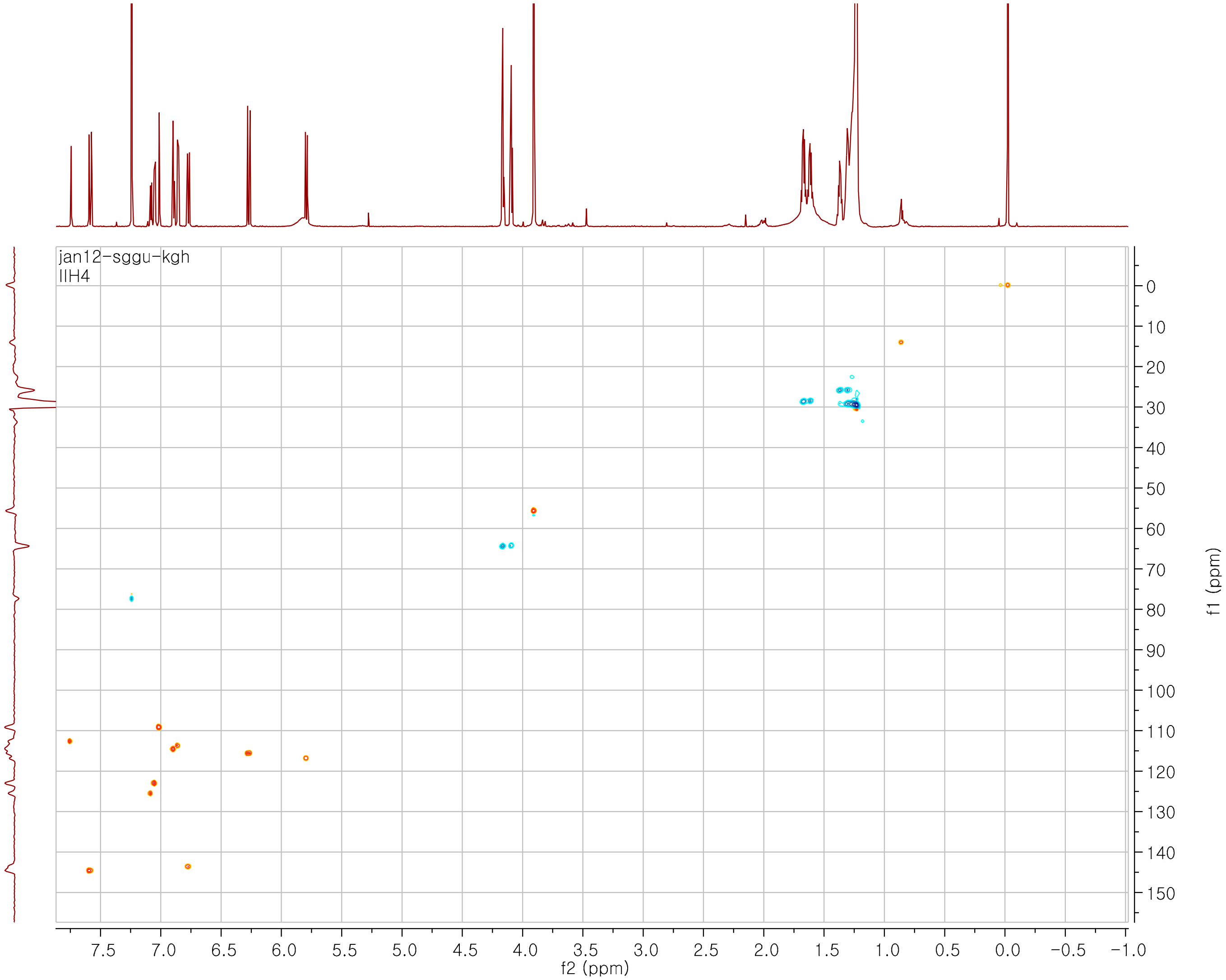

Figure S4. The HSQC spectrum of compounds **1** and **2.**

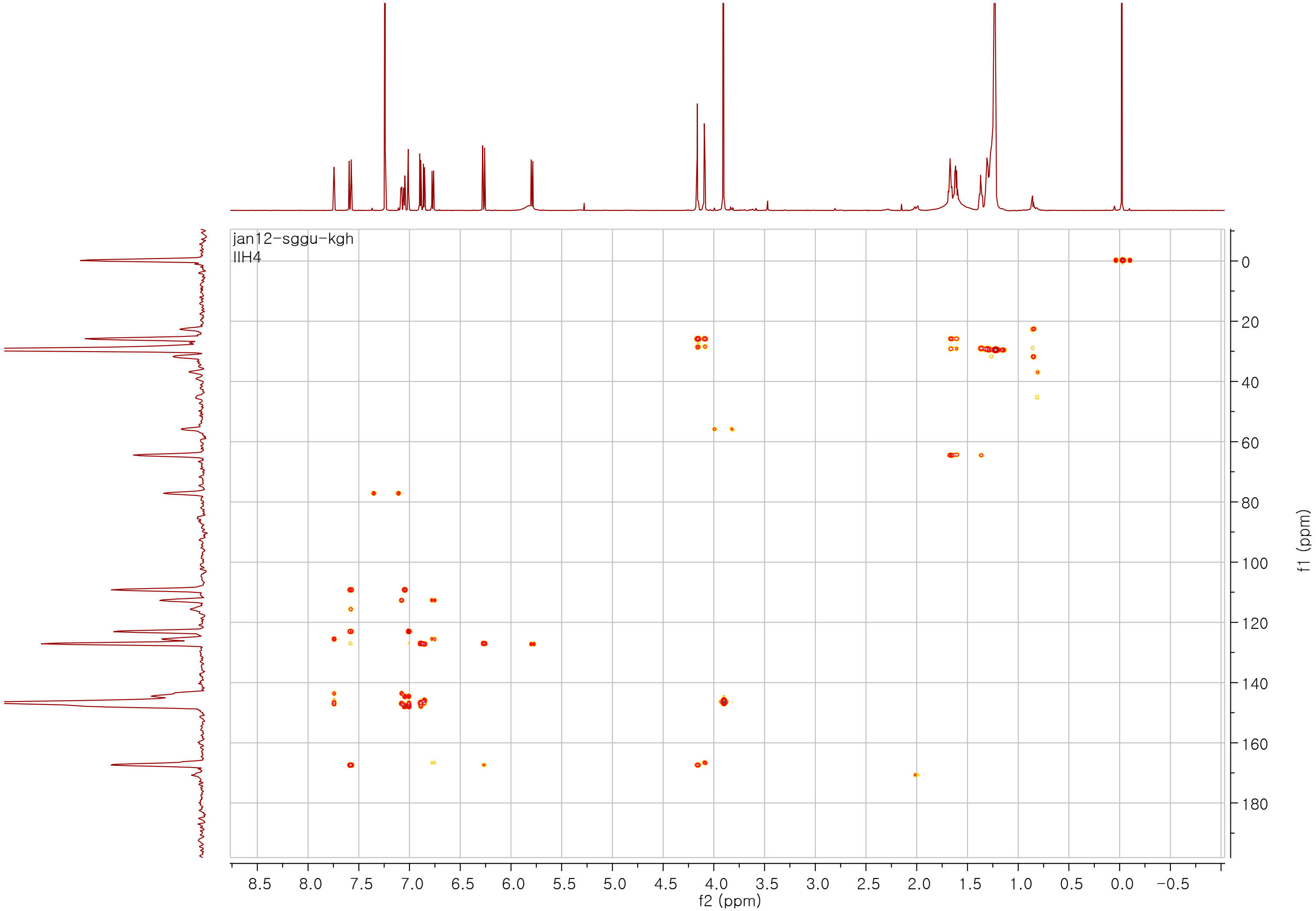

Figure S5. The HMBC spectrum of compounds **1** and **2.**
